# Longitudinal follow-up of zearalenone and deoxynivalenol mycotoxins in breast milk in the first five months of life

**DOI:** 10.1186/s40360-023-00677-8

**Published:** 2023-05-30

**Authors:** Bülent Güneş, Suzan Yalçın, Sıddika Songül Yalçın

**Affiliations:** 1 Şanlıurfa Training and Research Hospital, Child Health and Disease Service, Şanlıurfa, Turkey; 2 Department of Food Hygiene and Technology, Selcuk University Faculty of Veterinary Medicine, Konya, Turkey; 3 Department of Pediatrics, Faculty of Medicine, Hacettepe University, Ankara, Turkey

**Keywords:** Human milk, Mycotoxins, Zearalenone, Deoxynivalenol

## Abstract

**Objectives:**

There is a possibility for exposed lactating mammalians to transfer some contaminants to their milk. This study aimed to determine the levels and changes of Zearalenone (ZEN), Deoxynivalenol (DON) mycotoxins for the first five months in human milk.

**Methods:**

Voluntary lactating mothers having infants with gestational length ≥ 37 weeks were enrolled between August 2017 and June 2018 in Şanlıurfa. Mothers and infants with chronic health problems were not included in the study. Human milk samples were taken at three different times; on enrollment (Day 6–10, visit 1), between 4 and 6 weeks postpartum (visit 2), and between 14 and 19 weeks postpartum (visit 3). Mycotoxin levels in human milk were measured utilizing Helica brand commercial kit.

**Results:**

Nineteen voluntary mothers and their breastfed infants with three human milk samples completed the study. The mean ages of mothers and infant (± SD) were 27.4 (± 5.4) years and 7.6 (± 0.9) days on enrollment. Median levels of ZEN and DON in human milk samples were 0.39 and 16.7 ng/mL, respectively. None of the cases had a ZEN daily intake higher than 250 ng/kg bw per day. However, three fourth of the cases had DON intake higher than > 1000 ng/kg bw per day. When adjusted for infant weight for age and sex, both ZEN levels and daily intake were decreased progressively from visit 1 to visit 3 (p < 0.001). DON levels and daily intake at visit 2 were found to be significantly lower in samples of visit 3 than that taken in visit 2 (p = 0.004 and p < 0.001, respectively).

**Conclusions:**

Breast milk monitoring study revealed that ZEN and DON mycotoxins were present in the mother-infant environment. Contamination levels changed during the lactation period.

## Introduction

Mycotoxins are toxic metabolites produced by certain species of fungi and continue to attract global attention because of their impact on animal and human health. The most common mycotoxins in foods include aflatoxins; fumonisins; patulin; ochratoxin A; deoxynivalenol (DON); and zearalenone (ZEN) [1, 2].

Previous studies have addressed the toxic effects of ZEN and DON mycotoxin contaminants in animal models, including piglets, sheep, and rats. ZEN, as an estrogenic pollutant in mammalians, may cause several reproductive problems; impairment of folliculogenesis with impairment of primordial follicle formation in the ovaries, reduce sperm quality, delay in reproductive period, epigenetic changes, fetal reabsorption, increasing the frequency of stillbirths, low birth weights, and feminization of immature males [3–6]. ZEN produces vulvar dilatation, redness, and rectal prolapse. Additionally, ZEN is suggested to be a risk factor for precocious puberty and breast cancer in humans [7]. It was reported that DON may lead to vomiting and failure to thrive, immune dysregulation, leukopenia, and skin necrosis [8].

Mammalians are exposed to ZEN and DON by consuming different foodstuffs [9–12]. ZEN and DON are the most frequently detected mycotoxins in barley, sorghum, maize, wheat, rye, and other grains. Mycotoxins are metabolized after being taken into the body, the original forms or biotransformed structures of mycotoxins can be excreted in the urine. [13, 14]. Lactating mammals can transfer a small amount of exposed pollutants to their offspring through their milk [15]. Breast milk is one of the best matrices for biomonitoring studies because sufficient quantities of samples from the required sample size can be collected non-invasively. However, there are limited studies on the presence and levels of ZEN and DON mycotoxins in human milk [15–23]. Indeed, there is only one published study evaluating the presence of ZEN and DON in a longitudinal study [19] and this study included only one lactating woman.

Breastfeeding women can come into contact with ZEN and DON mycotoxins from foods they consume [24]. This study was performed to determine the presence and levels of ZEN and DON in human milk for the first five months in a cohort study. If mycotoxin carry-over is detected in breast milk in our study, both the mother’s diet and the choice of complementary foods for the baby can be controlled.

## Methods

For this cohort study, mothers with 6-10-day-old babies who applied to Private Şan Med Hospital Pediatrics Department in Şanlıurfa, Turkey between August 2017 and June 2018 were informed about the study.

Inclusion criteria for mother-infant pairs were: (a) Voluntary mothers without any known health problems including hypertension, diabetes mellitus, hypo/hyperthyroidism, (b) Infants whose gestational age ≥ 37 weeks, exclusively breastfeed (c) Mothers who planned to breastfeed for at least 6 months; (d) mothers enrolling to the hospital and giving milk samples at all the visits.

Exclusion criteria were (a) infants having congenital anomaly, (b) infants having mixed feeding, (c) infants from multiple pregnancies-twins, (d) mothers who stopped breastfeeding during the follow-up period.

The study protocol was approved by the Ethics Committee of Hacettepe University Faculty of Medicine for non-interventional studies. The mothers were informed and signed the consent form before the study.

On admission, characteristics of the mother (age, education, health problems, weight gain during pregnancy, weight, and height at birth) and infant (birth order, gestational length, birth type, sex, birth weight) were taken from the hospital file. Breast milk samples were taken on admission (visit 1; 6–10 days). They were invited for follow-up between 4 and 6 weeks (visit 2) and 14–19 weeks (visit 3). Maternal weight and infant weight, height, and head circumference were measured at all visits. Maternal body mass index was calculated. For infants z-scores of weight for age, height for age, weight for height, and head circumference for age (WAZ, HAZ, WHZ, and HCZ, respectively) were obtained from WHO Anthro software 3.2.

Dependent variables are levels of ZEN and DON in human milk.

To initiate the oxytocin reflex, the baby was breastfed for 5 min, then the milk sample was manually expressed and put into polypropylene tubes. They were stored at-20^o^C till the analysis was performed. Before analysis, 10 ml of milk was centrifuged at 3500 rpm for 10 min. After the fat layer was removed, the skim milk fraction were shifted to new tubes. Levels of ZEN and DON in human milk were determined using commercially available enzyme-linked immunosorbent assay (ELISA) kits (Helica, Catalog no 941DON01M-96, Catalog no 951ZEN01G-96). The assay was performed by manufacturer’s instructions.

The level to which the nursed infant (µg/kg bw per day) is exposed was calculated by multiplying the concentration in the milk (ng/mL) with the volume of milk consumed per day (L/kg bw per day) [25–27]. The amount of milk consumed by babies was estimated from EFSA data and 50% values were taken according to the baby’s age, gender, and body weight at the visit [25]. There is no published data for volume consumed by infants 6–7 days old and infants aged 112–140. Therefore, for these periods, the closest age range for which data was available in EFSA was taken (8–13 days for 6–7 and 84–111 days for 112–140) [25].

Statistical analyses were performed by SPSS 23.0 (SPSS INC., Chicago, IL) package program. Normality of data was controlled by Shapiro-Wilk test, kurtosis, skewness values and histogram. Normally distributed data were given as mean and standard deviations. Non-parametric tests were adopted because the distribution of the mycotoxin contamination data did not follow normality. The mean, quartiles, and 10 and 90 percentile values were identified for mycotoxins. The relationship between mycotoxin levels and continuous parameters of mother-infant pairs was examined by Spearman correlation analysis. Mycotoxin differences according to categorical mother-infant parameters were analyzed with Generalized Linear Models. Parameters showing p value < 0.2 in univariate analyses were taken for further analysis. Given the interaction among birth weight, WAZ, and HAZ we selected WAZ for binary analysis. Generalized estimating equations evaluated longitudinal changes in mycotoxin levels in milk with infants’ sex, birth order, and weight for age. Pairwise contrast was performed with the least significant difference (LSD). P < 0.05 was considered significant.

## Results

Twenty-six mother-infant pairs were enrolled, 2 mothers stopped breastfeeding and 5 mothers did not admit to control visits. Nineteen mothers meeting the study criteria and giving milk samples were included in the study.

The mean ages of mothers and infants were 27.4 years and 7.6 days on admission (Table 1). All mothers were housewives. Of all 2/3 of them had undergone cesarean delivery and 42.1% of them were primipara. 72.2% of babies were male.

**Table 1:** Characteristics of mother-infant pairs on admission and during follow-up period, n = 19*

	Visit 1 (On admission)	Visit 2	Visit 3
Postpartum days	7.6 ± 0.9	38.1 ± 7.0	122.4 ± 11.9
Maternal age, yrs	27.4 ± 5.4		
Maternal education ≥ 8 years, %	47.4		
Smoking status, %			
No smoke exposure or smoking	36.8	36.8	26.3
Maternal smoking +/-environmental smoke	10.5	15.8	26.3
Environmental smoke w/o maternal smoking	52.6	47.4	47.4
Maternal weight gain during pregnancy, kg	11.7 ± 6.2		
Maternal body mass index at birth, kg/m^2^	31.7 ± 4.2		
Maternal body mass index at visits, kg/m^2^	29.6 ± 4.1	29.0 ± 4.2	28.8 ± 4.2
Birth order, first child, %	42.1		
Birth type, cesarean delivery, %	63.2		
Gestational length, weeks	38.8 ± 1.0		
Birth weight, gr	3294 ± 517		
Infant sex, male, %	73.7		
**Infant anthropometry**			
Weight for age, z score	-0.21 (-0.73, 0.18)	-0.21 (-1.02, 1.02)	-0.06 (-0.82, 1.00)
Height for age, z score	-0.15 (-0.68, 0.46)	-0.83 (-1.33, -0.18)	-0.16 (-0.95, 0.56)
Weight for height, z score	-0.83 (-1.37, -0.03)	0.44 (-0.51, 1.23)	0.10 (-0.51, 1.19)
Head circumference for age, z score	-0.13 (-0.46, 0.69)	-0.32 (-1.34, 0.41)	-0.36 (-1.20, 0.33)
**Breast milk**			
Zearalenone, ng/mL	0.60 (0.35, 0.84)	0.43 (0.37, 0.49)	0.33 (0.26, 0.38)
Zearalenone, ng/kg bw per day	118 (69, 159)	81 (75, 99)	53 (41, 62)
Zearalenone > 0.25 µg/kg bw per day	0.0	0.0	0.0
Deoxynivalenol, ng/mL	16.7 (4.7, 25.8)	18.7 (12.5, 31.8)	11.3 (5.9, 18.1)
Deoxynivalenol, ng/kg bw per day	3273 (921, 4876)	3553 (2538, 6110)	1831 (956, 2841)
Deoxynivalenol > 1.0 µg/kg bw per day	73.7	89.5	73.7

*Data were given as % or mean ± SD or median (quartiles)

Median levels of ZEN and DON in all studied human milk samples were 0.39 ng/mL and 15.0 ng/mL, respectively (Table 2). The estimated intake was 0.189 µg/kg-bw per day for ZEN and 2.94 µg/kg-bw per day for DON. All ZEN levels were below 0.25 µg/kg bw per day (Table 1). However, more than three-fourths of samples had DON more than 1.0 µg/kg bw per day.

**Table 2:** Zearalenone and Deoxynivalenol concentrations and estimated intakes for 57 breast milk samples from 19 mothers

	Mean	Min	Percentiles					Max
			10	25	50	75	90	
Zearalenone,								
ng/mL	0.48	0.23	0.25	0.32	0.39	0.60	0.87	1.27
µg/kg-bw per day	0.183	0.157	0.161	0.162	0.189	0.196	0.203	0.214
Deoxynivalenol,								
ng/mL	16.4	1.0	3.5	6.9	15.0	24.5	33.0	37.4
µg/kg-bw per day	3.05	0.16	0.58	1.22	2.94	4.54	6.28	7.59

**Fig. 1:**
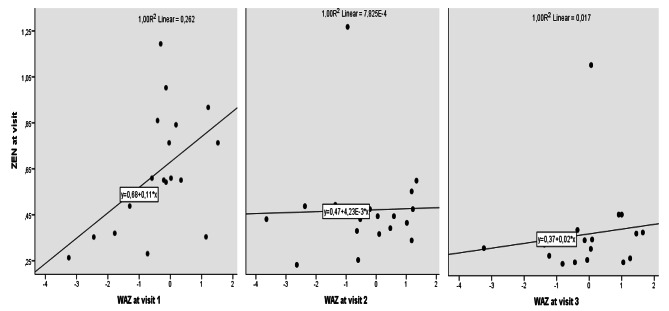
Scatterplot of Zearalenone (ZEN) with z score of weight for age (WAZ) at visits

When all milk samples were taken for analysis, there is no correlation between ZEN and DON (n = 57, r = 0.32, p = 0.17). Among mother-infant parameters, birth weight, infants’ WAZ and HAZ were found to be positively correlated with ZEN on admission (Table 3; Fig. 1). However, no interactions were detected at visit 2 and visit 3. Female infants had higher mean ZEN levels than male infants (Table 4). During follow-up period, ZEN levels decreased statistically. DON level was similar in the first two visits but decreased in the last visit. There were no differences in ZEN and DON levels according to other studied parameters (Tables 3 and 4).

**Table 3:** Correlations between mycotoxins and mother-infant parameters at visits*

	Zearalenone			Deoxynivalenol		
	Visit 1	Visit 2	Visit 3	Visit 1	Visit 2	Visit 3
Deoxynivalenol	0.30	0.16	0.11			
Maternal age	0.00	0.08	-0.10	-0.06	-0.01	-0.19
Maternal weight gain during pregnancy	0.17	-0.15	-0.18	-0.00	-0.00	-0.23
Maternal BMI at birth	-0.28	0.34	-0.32	-0.05	0.28	0.21
Maternal body mass index at the visit	-0.22	-0.26	-0.38	-0.15	0.37	0.19
Gestational length	0.03	0.23	0.25	0.12	0.21	-0.05
Postpartum day at the visit	0.29	-0.23	-0.11	0.25	-0.17	0.35
Birth weight	**0.49** ^**a**^	0.13	0.32	0.25	0.01	0.16
Weight for age, z score, at the visit	**0.55** ^**b**^	0.21	0.32	0.35	-0.00	0.10
Height for age, z score, at the visit	**0.59** ^**c**^	0.19	0.28	0.33	0.01	-0.06
Weight for height, z score, at the visit	0.14	0.13	0.12	0.04	0.03	0.21
Head circumference for age, z score, at the visit	0.41	0.03	0.13	0.41	0.13	0.06

* Spearman’s rho correlation Coefficient; ^a^p=0.032; ^b^p=0.016; ^c^p=0.007

**Table 4:** Differences in Zearalenone and Deoxynivalenol levels in all studied breast milk by some mother-infant parameters*

	n	Zearalenone, ng/mL		Deoxynivalenol, ng/mL	
		Mean [95% Wald CI]	p	Mean [95% Wald CI]	p
Sampling time			0.001		0.021
Visit 1	19	0.62 [0.52–0.72]^a^		16.4 [12.0-20.8]^ab^	
Visit 2	19	0.46 [0.36–0.56]^b^		20.8 [16.4–25.2]^a^	
Visit3	19	0.37 [0.27–0.47]^b^		12.0 [7.6–16.4]^b^	
Maternal education			0.663		0.236
< 8 years	30	0.47 [0.38–0.56]		14.9 [11.2–18.5]	
≥ 8 years	27	0.50 [0.41–0.59]		18.1 [14.2–22.0]	
Smoking status			0.435		0.539
No smoke exposure or smoking	19	0.45 [0.34–0.55]		17.8 [13.2–22.4]	
Maternal smoking +/-environmental smoke	10	0.57 [0.42–0.72]		13.3 [6.9–19.7]	
Environmental smoke w/o maternal smoke	28	0.48 [0.39–0.57]		16.5 [12.7–20.4]	
Birth order			0.819		0.170
First child	24	0.48 [0.38–0.57]		14.2 [10.1–18.3]	
≥ second child	33	0.49 [0.41–0.57]		18.0 [14.5–21.5]	
Infant sex			0.008		0.593
Male	42	0.44 [0.37–0.51]		16.8 [13.7–20.0]	
Female	15	0.62 [0.50–0.73]		15.2 [9.9–20.1]	

CI: Confidence Interval

*Generalized Linear Models for each parameters; ^a,b^ Groups with different letters are statistically different from each other; p < 0.05

When sex, birth order, and WAZ were analyzed with visit time, generalized estimating equations revealed an interaction for ZEN with sampling visit time and WAZ of infants. However, DON is associated with only sampling time (Table 5).

**Table 5:** Determinants of changes in Zearalenone and Deoxynivalenol levels in breast milk during lactational period*

	Zearalenone, ng/mL			Deoxynivalenol, ng/mL		
	Mean [95% Wald CI]	Wald Chi-Square	P*	Mean [95% Wald CI]	Wald Chi-Square	P*
Intercept		126.69	< 0.001		56.19	< 0.001
Sampling time		24.91	< 0.001		10.88	0.004
Visit 1	0.67 [0.58–0.76]^a^			15.8 [10.7–20.8]^ab^		
Visit 2	0.51 [0.38–0.63]^b^			20.1 (14.2–25.9]^a^		
Visit 3	0.40 [0.28–0.52]^c^			11.1 [6.6–15.6]^b^		
Sex		3.72	0.054		0.22	0.641
Male	0.44 [0.39–0.49]			16.6 [13.5–19.8]		
Female	0.61 [0.44-0.708]			14.6 [6.9–22.4]		
Birth order		0.04	0.842		1.24	0.265
First child	0.53 [0.38–0.68]			13.9 [8.3–19:6]		
≥ second	0.52 [0.46–0.58]			17.4 [12.8–21.9]		
Weight for age, z score		5.86	0.015		1.09	0.297

*Generalized Estimating Equations involving sampling time, sex, birth order, WAZ.

^a,b,c^ Groups with different letters are statistically different from each other; p < 0.05

CI: Confidence Interval

## Discussion

We found detectable levels of ZEN and DON mycotoxins in breastmilk. Previous studies in two provinces from Turkey reported that median ZEN and DON levels were 0.36 ng/mL and 8.55 ng/mL in Ankara [18], and 0.17 ng/mL (0.04 and 0.68 ng/mL) and 3.92 ng/mL (0.40-14.99 ng/mL) in Eskişehir, respectively [17]. Similarly, the ZEN level in human milk of 47 primipara mothers was between 0.26 and 1.78 ng/mL in Italy [28]. These studies were performed with ELISA. One study in Spain evaluated 35 breast milk samples (postpartum 30 days) with QuEChERS/UHPLCHRMS and reported the mycoestrogen ZEN and some of its metabolites in 13–67% with levels varied from 2.1 to 14.3 ng/mL and only one case having DON metabolite, DOM [21]. In another study, ZEN levels in the milk were 2.0–17.0 ng/mL in only 4% of 275 mothers having coeliac and 2.5–21.9 ng/mL in 8% of 178 healthy mothers [29]. A trace amount of ZEN was detected in pooled breast milk sample taken from the Austria milk bank [19]. An Iranian study reported that ZEN levels in none of the breast milk samples from 90 mothers were above the detection limit (≥ 0.005 ng/mL) with the HPLC method [23]. DON was not detected in breast milk in India [30]. The ZEN and DON detection in breast milk is found to be quite variable. One reason may be that climatic conditions of the studied environment or the region differently affect the fungal species and mycotoxin production [1, 22, 31]. Another reason might be related to food processing and the type of food eaten by the mother [1]. Besides, sampling methods, differences in analytical methods, and assay limits make it difficult to compare with other results [15]. Cross-reactivity reactions (ELISA) or interfering peaks (LC-FD) may be interpreted as positive signals [28]. In addition, postpartum day of milk sampling could affect mycotoxin levels. We detected changes in ZEN and DON levels with lactational duration.

Positive associations for ZEN with both birth weight and infant weight on Days 6–10 were detected in Şanlıurfa. This could be explained by the mycoestrogenic characteristics of ZEN. The interaction was observed in the early postnatal period. Similarly, infants (aged 0.5-4 months) with breast milk greater than 0.50 ng/mL had higher WAZ than their counterparts [32].

In our study in Şanlıurfa, a very weak correlation was found between ZEN and DON, no correlation was found in Ankara, and a strong correlation was found in Eskişehir (r = 0.622, p < 0.001) [17, 18]. This indicates that the exposure sources for ZEN and DON differ according to regions and climates.

To date, food regulatory authorities around the globe have not set permissible limits for the ZEN, and DON in any milk [33]. The EFSA Panel on Contaminants in the Food Chain derived a Tolerable Daily Intake (TDI) for ZEN of 0.25 µg/kg-bw based on a NOEL of 10 µg/kg-bw per day for estrogenic effects in female pigs with an uncertainty factor of 40 [2, 34]. The provisional group TDI value for DON plus 3-ADON and 15-ADON was 1.0 µg/kg bw/day [34]. Similar to previous studies in Turkey and Italy [17, 28], we calculated estimated intake of ZEN in human milk as 0.189 µg /kg/day, which is below 0.25 mg/kg-bw/day. The median estimated TDI value for DON was 2.94 µg /kg/day in human milk from Şanlıurfa, which is higher than Eskişehir study (0.75 µg/kg/day). In addition, 78.9% of 57 milk samples in the current study in Şanlıurfa were higher than 1.0 µg/kg bw/day. However, this is 36% in Eskişehir [17].

### Strengths and limitations

Although limited by small sample size (n = 19), ZEN and DON level changes in the first six months, consisting of 3 periods, were analyzed for the first time. The longitudinal cohort study in breast milk samples showed changes in exposure to mycotoxins during lactation. Due to the small number of cases, its generalizability is limited. However, previously, only one mother followed for one year [19]. Male predominance was a selection bias in the study as a limitation. Other limitation is that the toxin concentration determinations were performed with EFSA data [25]. There, whereas, due to the absence of estimated data for food intake for the enrolled age groups, we calculated intakes for closest age range. The potential errors in the intake estimates might be occurred. In the evaluations, the error was tried to be minimized by using body weight and gender. Another limitation is that ELISA was used for toxin detection when already better methods like HPLC/LCMS are available, which are more accurate and reliable with better sensitivity and specificity [1].

Previous studies have highlighted the high mycotoxin exposure of weaned infants compared to breastfed infants [9, 12, 35]. Similarly, the detection frequency and median concentrations were found to be greater in urine of non-exclusively breastfed compared to exclusively breastfed infants (83 vs. 57% for ZEN; 55 vs. 30% for DON; 0.10 vs. 0.08 ng/mL for ZEN, 2.13 vs. 0.44 ng/mL for DON) [16]. In addition, the maternal dietary habits and the consumption of contaminated foods, and the presence of mycotoxin around mother and infant environment might influence the contamination of breast milk. Therefore, breast milk is significantly less harmful to infants as compared with other dietary sources that may be contaminated at higher concentrations. Moreover, new benefits of breast milk for the development and health of babies are being discovered every day.

In conclusion, mycotoxins including ZEN and DON may pass from some pollutants which are present around mothers and babies to breast milk. However, their levels decreased during lactation age. The fact that we determined the change in the mycotoxin amount in different lactation stages shows that infant age should be taken into consideration when organizing a new study. Detection of ZEN and DON mycotoxins in breast milk, even in small amounts, indicates its presence in the foods consumed by the lactating mother. This situation requires attention in the selection and preparation of food for both the lactating mother and the infant during the complementary feeding stage. There is a need for studies that take into consideration the mother’s food choice and that include different regions, which will provide information about the prevalence of mycotoxin.

## Data Availability

Data can be requested from corresponding author (siyalcin@hacettepe.edu.tr).
